# Chronic recurrent multifocal osteomyelitis: follow up Longitudinal case series study for five years with radiographic and scintigraphic imaging perspective and health outcome analysis with EQ5D-5L

**DOI:** 10.1186/1546-0096-13-S1-P188

**Published:** 2015-09-28

**Authors:** N Roy, S Sathyanandan, K Lalitha, M Mathew

**Affiliations:** 1 Medical College Trivandrum, Department of Radiodiagnosis and Imageology, Trivandrum, India; 2 Medical College Trivandrum, Department of Pediatrics, Trivandrum, India; 3 Tata Memorial Centre, Department of Immunohematology & Transfusion, Mumbai, India

## Introduction

Chronic recurrent mutifocal osteomyelitis (CRMO) (OMIM-number-259680), a rare form of autoinflammatory disease of the bone marrow of unknown aetiology, which takes a sub-acute course. First described in 1972 by Gideon et al, CRMO is primarly a diagnosis of exclusion as suggested by negative bone biopsy for neoplasms and negative bone culture to rule out infectious causes. In our case, we have a case cohort of a female child from 2011 until the present. During each episode of the disease, NSAIDs, empirical antibiotics and a course of steroids were given.

## Objectives

### Primary objective

To identify the radiographic imaging perspective of the recurrent inflammatory attacks on the bone, considering time period of each attack, a follow up case cohort for five years.

### Secondary objective

Elucidation of existing proposed diagnostic (King et al and Iyer et al) criteria & utility in aiding diagnosis as to the imaging features and validation of generic health outcome of a CRMO patient with EQ-5D 5L

## Methods

Study design: A longitudinal case series study.

Study setting: Trivandrum Medical College.

Study period: 18 May 2010 – Ongoing.

Study subjects: Patient and relevent family members with genotypic variance.

Study tool: EQ-5D-5L, Descriptive system of health related quality of life states in 5 dimensions by EuroQoL with permission.

## Results

The first presentation was at 9 years of age with a clinical lesion on the right 2nd metatarsal. X-ray revealed hypolucent lytic lesion. A bone biopsy for histopathology revealed chronic inflammatory infiltrate with predominant monocytes & lymphocytes suggestive of osteomyelitis, bacteriological culture of the sample was sterile and mycobacterial-RT-PCR was negative.

The second episode was after two weeks at the right-shoulder and X-ray revealed a permeative sclerotic lesion at humerus. PDFS, T1WI and T2WI MRI shows hypointense rim was suggestive of sclerosis with contrast enhancement suggestive of subperiosteal infection. Investigations included negative HLA-B27, negative ANA-profile for screening of autoimmune disorders, negative CRP and ESR showed elevation. Abdominal-ultrasound to rule out IBD and was normal. MRI was taken & provisional diagnosis of CRMO was made based on Iyer et al criteria.

In 2015, recurrence of pain occurred in the right arm. 3-phase-Tc99MDP whole-body-scintigraphy did not show any asymptomatic foci of infection other than the humerus. A follow up MRI showed that the lesion has extended distally towards the diaphysis of humerus 23cm down towards elbow, when compared to the previous year imaging. EQ5D-5L index value as generic health outcome assessment is 0.722, with Zimbabwe taken arbitarily as standard. (max value is 1 assigned for positive health), EQ-VAS scale coded response value in is 72. (max value for best possible state of health is 100).

## Discussion

The radiographic pattern, shows that TIRM, STIR sequence is helpful in assessing marrow edema, diffusion restriction is not seen usually, CT scan aids in assessing cortical thickening of bone and post contrast enhancement of lesion present indicative of a severe disease. A 3-phase-bone-scintigraphy, help to pick asymptomatic sites(sensitivity 73% to 100%) usually shows uptake in all three phases

As per Angela et al, the effect of attenuated TLR4/MAPK signalling & IL-10 polymorphism with reduced Sp1 recruitment & attenuated H3S10 phosphorylation contributes to central pathophysiology of CNO. Hence satisfying Iyer et al or King et al criteria alone does not serve as a platform to start on a patient with immunomodulatory therapy, cytogenetic profiling is advised prior to its commencement. The serum level of calcium shows hypocalcaemia, hypophosphatasia was not attributed in this case. TPMT assay is withheld considering financial condition of patient. Treatment protocol with anti-TNFα inhibitors & other immunomodulatory therapy is initiated for severe disease with low heath outcome. immunomapping of chromosome 18q21.3, an assessment of 1L10, TNFα, IL-1β, and LPIN2 mutation in (Majeed Syndrome), TNSALP gene mutation (metabolic defect with hypophosphatasia involving the NLRP3 inflammasome) are evidence based optional cytogentic tests. Dysregulation in sex hormones in CRMO are being investigated.

## Conclusion

Radiographic imaging (MRI) & whole-body-bone scintigraphy should be preferably used as initial diagnostic modalities for screening and follow up. Standardised imaging analysis as compared to existing proposed clinical criteria (King et al, Iyer et al etc) can only aid in establishing diagnosis. Health outcome assessment using EQ5D-5L with crossover with EQ5D-3L index value which shows moderate satisfactory outcome.

